# Why study plasticity in multiple traits? New hypotheses for how phenotypically plastic traits interact during development and selection

**DOI:** 10.1111/evo.14464

**Published:** 2022-03-20

**Authors:** Matthew E. Nielsen, Daniel R. Papaj

**Affiliations:** ^1^ Department of Ecology and Evolutionary Biology University of Arizona Tucson Arizona 85721; ^2^ Zoology Department Stockholm University Stockholm 11419 Sweden

**Keywords:** Cue, multivariate plasticity, phenotypic plasticity, reaction norm, reversible plasticity

## Abstract

Organisms can often respond adaptively to a change in their environment through phenotypic plasticity in multiple traits, a phenomenon termed as multivariate plasticity. These different plastic responses could interact and affect each other's development as well as selection on each other, but the causes and consequences of these interactions have received relatively little attention. Here, we propose a new conceptual framework for understanding how different plastic responses can affect each other's development and why organisms should have multiple plastic responses. A plastic change in one trait could alter the phenotype of a second plastic trait by changing either the cue received by the organism (cue‐mediated effect) or the response to that cue (response‐mediated effect). Multivariate plasticity could benefit the organism either because the plastic responses work better when expressed together (synergy) or because each response is more effective under different environmental circumstances (complementarity). We illustrate these hypotheses with case studies, focusing on interactions between behavior and morphology, plastic traits that differ in their reversibility. Future empirical and theoretical research should investigate the consequences of these interactions for additional factors important for the evolution of plasticity, such as the limits and costs of plasticity.

The environment of any organism varies in both space and time. Phenotypic plasticity, when a single genotype expresses different phenotypes in different environments, is a key means by which organisms respond to environmental variation (DeWitt and Scheiner 2004; Ghalambor et al. 2007). An organism interacts with this variation twice: first the environment alters the development of a trait, then the environment selects on that trait (Moran 1992). How the environment alters development can be described using a reaction norm, a function that relates the state of the environment to the phenotype produced in response (Woltereck 1909). Here we focus on adaptive plasticity, which occurs when, over an appropriate range of environments, the plastic genotype has higher average fitness than nonplastic genotypes (Scheiner 1993). Adaptive plasticity can occur in many types of trait, from changes in gene expression and biochemistry to physiology and morphology to behavior and life history (Stearns 1989; Foster et al. 2015). As such, the evolutionary consequences of plasticity have been discussed for more than a century (e.g., Baldwin 1896) and have received particular attention in recent decades (e.g., Stearns 1989; West‐Eberhard 2003, Ghalambor et al. 2007, Hendry 2016; Pfennig 2021).

Although phenotypic plasticity is commonly studied on individual traits in isolation (Stamp and Hadfield 2020), organisms frequently respond to environmental change with adaptive plasticity in multiple traits (Schlichting 1989a; Relyea 2004; Foster et al. 2015). Some of the best studied cases of such multivariate plasticity concern induced defenses to predators by aquatic animals: prey can alter multiple aspects of their behavior, morphology, and life history when predators are present in their environment (Spitze and Sadler 1996; Relyea 2004; Kishida et al. 2010). As another example, many semiaquatic plants display heterophylly, where their leaves above and below water are shaped differently, but they also change other characteristics of underwater leaves, such as reducing cuticle thickness and the density of stomata (Wells and Pigliucci 2000).

If we focus on only a single trait when studying plasticity, we may miss plastic changes that occur in other traits and might thus reach misleading, or even incorrect, conclusions about plasticity in the focal trait (e.g., Spitze and Sadler 1996). Just as the environment interacts with plastic traits in two different ways, development and selection, plastic traits can potentially interact with each other during each of those same processes. Studies of the phenotypic integration of plasticity demonstrate the interdependence of the development of plastic traits by showing that plastic responses in different traits can be correlated with each other (Box 1). The importance of multivariate plasticity for selection is indicated by models where incorporating plasticity in multiple traits changes the optimal response of plastic traits to the environment and the maximum fitness that can be achieved, even without any explicit interaction between the traits (Steiner and Pfeiffer 2007). The correlative approach used to study phenotypic integration has provided considerable insight into multivariate plasticity, but alternative approaches are needed to address causal relationships among plastic traits (Tonsor and Scheiner 2007). Here, we introduce a complementary approach which uses manipulative experiments to explore the causal relationships between traits. We focus on behavior and morphological traits because they present some of the best existing examples and often change on distinctly different time scales (Foster et al. 2015) which facilitates their independent manipulation.

**Box 1: Phenotypic integration and plasticity**

Phenotypic integration has been the primary approach used to study the relationships among multiple traits. It describes the co‐expression of traits; specifically, two traits are considered phenotypically integrated when their values are correlated with each other (Pigliucci 2003). The idea of phenotypic integration has its origins in the study of morphology (Olson and Miller 1958; Cheverud 1996); however, it can be applied to traits of any kind. For example, the study of behavioral syndromes, a major topic in contemporary behavioral ecology, emphasizes correlations among different behaviors in different contexts (Sih et al. 2004), essentially the phenotypic integration of behavior.

Historically, the integration and plasticity of traits were often placed at odds with each other (Schlichting 1989a). Integration should constrain plasticity because the more closely connected a trait's expression is to other traits, the harder it would be for that trait to change in response to the environment, particularly in an adaptive way (Gianoli and Palacio‐López 2009). On the other hand, plasticity should constrain integration because plastic variation may obscure other sources of phenotypic variation, and correlations between traits should be reduced if genotypes differ in their plasticity for the two traits (Stearns 1989). Some empirical evidence exists for this negative correlation between integration and plasticity (Gianoli and Palacio‐López 2009); however, even if plasticity and integration often oppose each other, they can still occur among the same traits and can interact conceptually in two major ways. First is the plasticity of integration: in other words, the correlation between two traits may depend on the environment (Schlichting 1989a; Kasumovic 2013). Although plasticity can interfere with and decrease integration under some conditions, traits can instead become more integrated under other conditions. Second is the integration of plasticity: in other words, the degree of plasticity of different traits be correlated (Schlichting 1989a). Integration of plasticity is a logical consequence of the fact that plasticity is a trait in and of itself and implies that the capacities of different traits to respond to an environmental change may be interrelated.

The phenotypic integration approach has historically dominated research on multivariate plasticity, concentrating on measuring the correlation among different traits in different environments (e.g., Schlichting 1989b; Waitt and Levin 1993; Nicotra et al. 1997; Boersma et al. 1998; Callahan and Waller 2000; Pigliucci and Hayden 2001; Relyea 2001; Pigliucci and Kolodynska 2002; Hoverman et al. 2005; Chun et al. 2007; Sánchez et al. 2007; Gianoli and Palacio‐López 2009; Husby et al. 2010; Montague et al. 2013; Lind et al. 2015). Although the environment in these studies is often experimentally manipulated, the traits themselves are not, leaving these studies agnostic to the actual cause of co‐expression between traits, outside of the shared environment. The reliance on correlation also limits the ability of these studies to answer questions about causal relationships among plastic traits.

Assessing causal relationships in multivariate plasticity can allow us to answer new questions about both the development of plastic traits and selection on those traits: how can plasticity in one trait alter the response of other plastic traits to an environmental change, and under what conditions would selection favor plasticity in multiple traits as opposed to just one? Answering these questions has important implications for classic topics in the study of the evolution of plasticity, including the costs of plasticity and the role of plasticity in facilitating survival in and adaptation to novel environments.

## Development of Plasticity in Multiple Traits: How do Plastic Traits Affect Each Other's Responses to the Environment?

When multiple traits of an organism are plastic, a change in one trait can potentially alter the development and expression of another. The plastic change in the first trait may simply increase or decrease the phenotype of the second trait in the same way across all environments, changing the intercept of the reaction norm. In the extreme case, the phenotype of the second trait may not respond directly to the environment at all but have its phenotype entirely determined by the first trait. Although models suggest this scenario is unlikely to be adaptive if noise is present (Scheiner 2018), this is one way to represent the “higher level” consequences of plasticity in hormones and other internal signals. Alternatively, the effect of the first trait may vary with the environment, creating a more complex, nonadditive change in the apparent reaction norm of the second trait. As an example, *Latrodectus hesperus* (black widow) spiders respond to variation in food availability via plasticity in two traits: the structure of their web and the speed with which they respond to vibrations that indicate prey (DiRienzo and Montiglio 2016). When spiders are moved to webs made by other spiders, the speed with which they attack prey changes based in part on the structure of the new web (Montiglio and DiRienzo 2016), showing that plasticity in one trait, web structure, can cause a change in a second plastic trait, foraging behavior.

**Figure 1:**
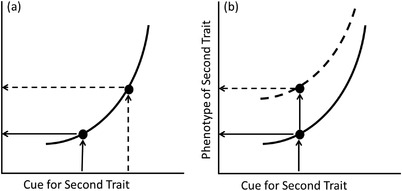
Diagram illustrating cue‐mediated and response‐mediated effects of a change in one plastic trait on the phenotype of a second. The reaction norm for the second trait is plotted with arrows showing how a cue is translated into a phenotype. The first trait itself is not shown, but solid and dashed lines represent the consequences of two different values of the first trait's phenotype for plasticity in the second trait. (a) *Cue‐mediated interaction*: a change in the first trait changes the value of the cue that the second trait responds to. This is shown by two different cues (solid vs. dashed arrows) as inputs into the same reaction norm, leading to two different outputs. (b) *Response‐mediated interaction*: a change in the first trait changes how the second trait responds to its cue, as described by a change in the apparent reaction norm (solid versus dashed curve). Now the same cue is input into two different reaction norms, leading to different outputs.

But how does plasticity in one plastic trait affect another trait's development? Here, we take a broad view of development (in the manner of West‐Eberhard 2003) that encompasses all changes in an organism over the course of its life span, including rapid or temporary ones such as the expression of a behavior. In general, a plastic change in a given trait occurs in two broad steps. First, the environment changes; specifically, a cue (the environmental factor which affects the development of the plastic trait) changes (Moran 1992). Second, the organism responds to the environmental change by changing its phenotype, as described by the reaction norm (Windig et al. 2004). For a given trait, both steps could be affected by the phenotypes of other traits, and thus plasticity in one trait could alter development of a second trait by affecting either the cue or the response to the cue (Table 1; Fig. 1). These two mechanisms are not mutually exclusive but can be distinguished by whether the cue received by the organism changes or the mechanisms producing the plastic response to that cue change.

**Table 1:** Nonmutually exclusive mechanisms by which a change in one plastic trait can alter the development of a second plastic trait

I. Cue‐mediated	Changing the first trait changes the state of the cue which the second trait responds to. Habitat construction: plastic change in the first trait physically or chemically alters the organism's environment, subsequently changing the cue received. Habitat choice: plastic change in the first trait changes what environment the organism is exposed to, subsequently changing the cue received. Direct cue modification: plastic change in the first trait directly changes the cue for the second trait as experienced by the organism, without changing the rest of the environment.	Examples: *Battus philenor*: body coloration changes body temperature, the cue for refuge‐seeking behavior. *Calidris canutus*: foraging ground choice determines prey hardness, the cue for gizzard size.
II. Response‐mediated	Changing the first trait changes how the second trait responds to its cue (reflected in a change in the reaction norm). Perception: changing the first trait changes how the organism detects or perceives the second trait's cue. Internal processing: changing the first trait changes how the perceived information is processed within the organism (e.g., via hormones or neurons). Phenotype production: changing the first trait changes how the second trait is formed in response to perceived and processed signals.	Examples: None yet identified.^a^

^a^ Although we do not have a clear empirical example of a response‐mediated interaction, both *B. philenor and C. canutus* provide examples of negative tests for response‐mediated interactions.

The environment experienced by an organism is fundamentally dependent on the organism itself (Lewontin 1983). Because organisms possess traits that affect their environment (Sultan 2015), if a plastic change in one of these traits alters the organism's environment in a way that affects the cues for another plastic trait, this will produce a *cue‐mediated interaction* between those traits. These changes in cues can occur through a variety of processes that vary in how much the environment overall changes (Sultan 2015). Organisms often alter their environment physically, chemically, or otherwise in lasting ways, a process known as habitat construction (Sultan 2015, also referred to as niche construction, Odling‐Smee et al. 2003, 2013). This habitat construction can then in turn alter the expression of plastic traits in the constructed environment (Saltz and Nuzhdin 2014; Moczek 2015). Even if organisms are not physically changing their environment, they can control which environments they are exposed to through habitat choice (Donohue 2005; Stamps 2006). Through their traits, organisms can also change how they experience their environment; for example, many aspects of morphology influence the temperature experienced by an organism (Sultan 2015). Although not plasticity in themselves, any of these mechanisms can depend on plastic traits, and so long as the changes an organism makes to its experienced environment also change the cue for a plastic trait—either directly or by changing a different aspect of the environment that in turn changes the cue—the change in the cue will also change the plastic trait's phenotype.

Independent of effects on the experienced cue, a change in one trait could cause a change in a second plastic trait by altering how the second trait responds to that cue, producing a *response‐mediated interaction*, reflected by an apparent change in the second trait's reaction norm. Adaptive plasticity occurs via a series of internal, physiological processes: perception (the organism in some way detects the state of the cue), internal processing (the perceived information is processed by the organism), and phenotype formation (the signal leads to the production of a different phenotype) (Windig et al. 2004). If plasticity in one trait alters one or more of these steps for a second trait, the apparent reaction norm for the second trait will likewise change. It may be particularly easy for a change in one trait to affect others in situations with physiological pleiotropy, where a single hormone or other physiological mechanism controls plasticity in multiple traits (Ledón‐Rettig and Ragsdale 2021), and shared or overlapping regulatory pathways may often underlie response‐mediated interactions. Testing for cue‐mediated or response‐mediated interactions relies on experimentally manipulating one trait and testing under what conditions the second trait changes (Box 2). Nevertheless, establishing causal interactions between plastic traits that respond to a shared regulatory or physiological mechanism may be particularly difficult because those same shared mechanisms will make independent manipulation more difficult. Instead, causation may be easier to detect in cases of serial developmental integration (sensu Lande 2019), where fixed or slowly reversible plasticity influences the expression of more quickly reversible traits.

**Box 2: Testing developmental interactions among plastic traits**

Whereas most previous work on multivariate plasticity has focused on correlation between traits (Box 1), the hypotheses presented here focus on causation. Thus, empirically testing these hypotheses requires new approaches. The ideal way to establish causation is the classic way: manipulative experiments. Altering the developmental environment provides a natural way to manipulate a plastic trait's phenotype, enabling these experiments (see Chevin et al. 2021, for a discussion of this approach in the context of relating gene expression to phenotype). For multivariate plasticity, however, it can be challenging to manipulate the responses of different traits independently. Independent manipulation will often be easier when traits differ in their reversibility or developmental timing, in which case varying the timing and duration of exposure to cues can separate the plastic responses of different traits.

Cue‐mediated and response‐mediated interactions represent two different pathways by which developmental interactions can occur, and one way to test them is to experimentally remove one of these pathways and observe if the interaction persists. When testing for cue‐mediated interactions, manipulating the environment such that changes in the first trait no longer change the cue for the second can remove this pathway. Any remaining interactions should be response‐mediated. For example, the cue‐mediated pathway was removed in *Battus philenor* by manipulating the light environment such that color could no longer affect body temperature, refuge‐seeking behavior's cue (Nielsen et al. 2018). Because this manipulation removed the effect of body coloration on temperature, it provided de facto evidence of a cue‐mediated interaction. In some cases, response‐mediated interactions can be removed by generating the plastic phenotypes of a trait artificially. This manipulation can circumvent the physiological processes that produce the plastic change, thus removing most pathways for changing the reaction norms of other traits. For example, artificial manipulation of body coloration using black paint or ink has been used in several insect species to demonstrate an effect of body coloration on thermoregulatory behavior (Kingsolver 1987; Karpestam et al. 2012). These experiments, of course, require careful controls for any physiological consequences of the phenotype manipulation, such as stress. Directly measuring the cue or reaction norm during an experiment and relating it to the manipulated trait can provide additional evidence regarding cue‐ or response‐mediated interactions. For example, the *B. philenor* study ruled out a response‐mediated effect of body color on refuge‐seeking by directly measuring part of the behavior's reaction norm and showing body coloration had no effect on it (Nielsen et al. 2018).

### EXAMPLES OF INTERACTIONS DURING DEVELOPMENT

*Battus philenor (*pipevine swallowtail) caterpillars provide an example of a cue‐mediated interaction between plastic traits. These caterpillars respond plastically to high temperatures by changing body coloration from black to red, which absorbs less solar radiation and makes them cooler, and by leaving their host plant to seek cooler locations as a thermal refuge (Nice and Fordyce 2006). Red coloration has been shown experimentally to reduce the frequency of refuge seeking under field conditions (Nielsen and Papaj 2017). Further lab research has shown that this interaction is cue‐mediated, occurring through the effect of color on body temperature (the cue for the behavior); under low‐light conditions in which color no longer affects body temperature, the interaction no longer occurs (Nielsen et al. 2018). An additional response‐mediated interaction was ruled out by showing that body color does not alter the body temperature threshold for refuge‐seeking (i.e., the behavior's reaction norm) (Nielsen et al. 2018).

This *B. philenor* example illustrates how morphology can alter behavior through a cue‐mediated interaction. Foraging by *Calidris canutus* (red knots), a molluscivorous shorebird, provides an example of a cue‐mediated interaction in the opposite direction, in which a change in behavior can alter morphology. These birds maximize foraging efficiency under different environmental conditions through changes in behavior, but also through changes in their digestive system. First, each day they choose a foraging site, with most sites being characterized by either a high abundance of hard‐shelled, hard‐to‐digest prey or a low abundance of soft‐bodied, easy to digest prey (van Gils et al. 2005). Over a period of weeks, they also adjust the size of their gizzard, with a larger gizzard allowing faster processing of hard‐shelled prey (Dekinga et al. 2001; van Gils et al. 2003). Diet provides the cue for gizzard size plasticity such that birds that were experimentally fed harder prey developed a larger gizzard (Dekinga et al. 2001; van Gils et al. 2003; Bijleveld et al. 2014; Mathot et al. 2017). Under lab conditions, individual variation in diet preference persists over extended periods, long enough to lead to changes in gizzard size through its effect on this dietary cue (Mathot et al. 2017), illustrating the potential for a cue‐mediated interaction. *Calidris canutus* also provides an opportunity to test for a response‐mediated interaction in the opposite direction: gizzard size could influence the behavioral response to cues used in foraging. Tracking of wild birds showed that birds with large gizzards choose sites with abundant, hard prey, while birds with small gizzards choose sites with scarce, soft prey (van Gils et al. 2005; Bijleveld et al. 2016). Experimental manipulation of gizzard size, however, showed that gizzard size has minimal if any effect on diet choice, which is instead determined primarily by individual variation independent of gizzard size (Mathot et al. 2017). Thus, gizzard size is unlikely to be altering how birds respond to the cues used in foraging, so a response‐mediated interaction is not present in this case.

## Selection on Plasticity in Multiple Traits: Why Have Multiple Plastic Traits?

**Figure 2:**
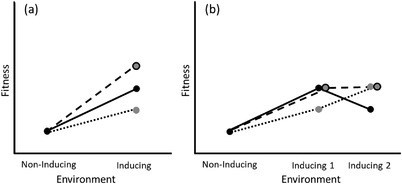
Diagram illustrating the synergy and complementarity hypotheses for the fitness benefit of having multiple plastic responses to an environmental change. Fitness is shown in the noninducing environment and inducing environment for two genotypes each with induced plasticity in a different trait (dotted and solid lines represent the two genotypes). Fitness is also shown for a genotype with plasticity in both traits (dashed line). (a) *Synergy hypothesis*: fitness in the inducing environment is greater when both plastic responses occur than for either response alone, shown by the greater fitness of the combined response (dashed line) than either individual response (solid or dotted). (b) *Complementarity hypothesis*: the fitness benefit of each plastic response is greater in a different inducing environment. Each plastic trait may be beneficial in all inducing environments, but trait one (solid line) provides a greater benefit in the first environment, while trait two (dotted line) provides a greater benefit in the second environment. An organism with plasticity in both traits (dashed line) can have high fitness in both environments even if no additional benefit is provided by expressing both traits simultaneously (i.e., no synergy).

For plasticity in a single trait to be adaptive, a population or individual must be exposed to environmental variation, and the optimal phenotype for that trait needs to vary among those environments (DeWitt and Scheiner 2004; Doughty and Reznick 2004). The same basic reasoning applies to adaptive multivariate plasticity; however, the benefits provided by the different traits may not be independent. Understanding the interdependence of selection on different plastic traits can help us assess the overall benefit of having a multivariate response to a given environmental change. Why should an organism respond to an environmental change with plasticity in multiple traits? This question is especially relevant when we consider that adaptive plasticity is not found in all traits (Palacio‐López et al. 2015; Acasuso‐Rivero et al 2019) and may be constrained by its costs and limits (DeWitt et al. 1998; Auld, Agrawal, and Relyea 2010). Plasticity is costly when a plastic individual has lower fitness than a less plastic individual with the same trait value (DeWitt et al. 1998; Auld, Agrawal, and Relyea 2010). Although costs of plasticity have been difficult to detect in many cases (Auld, Agrawal, and Relyea 2010), these costs may be greatest for the most flexible forms of plasticity (Snell‐Rood et al. 2018). If costs are not shared between plasticity in different traits, they should select for plasticity in as few traits as possible. Even if plasticity has minimal costs, plasticity can have a variety of limits which prevent it from producing an optimal phenotype (DeWitt et al. 1998). One of these limits, the reliability of information, may be particularly strong for multivariate plasticity because response‐mediated developmental interactions are predicted to interfere with the accuracy of responses to environmental cues (Scheiner 2018). Nevertheless, multivariate plasticity should evolve if changing multiple traits is in some way more feasible, more effective, or more efficient than making a greater change in a single trait. In general, multivariate plasticity can be beneficial in two, nonmutually exclusive ways: multiple plastic responses could provide a greater overall benefit when they occur simultaneously (*synergy*), or the different responses could each provide a greater benefit than the other under different ecological circumstances, even if they perform the same general function (*complementarity*) (Fig. 2). Each of these two broader hypotheses can apply in several ways (Table 2).

**Table 2:** Nonmutually exclusive hypotheses for the benefit of adaptive plasticity in multiple traits

I. Synergy	Plastic responses provide a greater benefit when changed together Functional integration: traits affect selection on each other, such that the performance benefit of changing both is more than additive Overcoming limits: changing a second trait overcomes limits to the expression or plasticity of another.	Examples: *Hyla chrysocelis*: reduced activity reduces predation risk more for tadpoles with a morphological response (deeper tail).
II. Complementarity	Each plastic response provides a greater net benefit in different conditions. Will involve some combination of the following: Differing benefits: each response increases performance more under different environmental conditions Differing costs: costs and tradeoffs associated with each trait vary, so each response is better suited to different environments, particularly resource availability Differing reversibility: plastic responses vary in degree or rate of reversibility, so that each response is better suited to different rates of environmental change	Examples: *Rana temporia*: foraging costs of activity reduction to avoid predators greater at high conspecific densities, so greater use of morphological plasticity at high density. *Battus philenor*: plasticity of body coloration is slower than refuge‐seeking behavior but avoids its costs.

The *synergy hypothesis* predicts that plastic responses will increase performance more when changed together than when either response occurs alone. Synergy can result if the plastic traits are functionally integrated (i.e., their effect on performance and fitness depends on each other). Functional integration constrains the evolution of nonplastic traits so that a genetic change in one trait is typically associated with a change in the other (Cheverud 1996 **;** Schwenk and Wagner 2001). The same reasoning can be extended to plastic traits, and if a response to an environmental change is more effective when multiple traits are changed, plasticity should evolve in both these traits. Even if traits are not functionally integrated, synergy can also result if the plasticity of individual traits is limited. Limitations on the phenotypic range of a plastic trait can create an important constraint on plasticity (DeWitt et al. 1998), and the ideal phenotype for an environment can sometimes be impossible to achieve via either plasticity or genetics. When this occurs, additional change in a second trait can overcome this limitation and improve performance even if the benefits provided by the plastic changes are independent. In all cases of synergy, performance and fitness should be greater when the plastic changes are made simultaneously than when either change occurs separately (Fig. 2a).

In contrast to the synergy hypothesis, the key condition for the *complementarity hypothesis* is that different environmental conditions favor each plastic response, such that averaged across these conditions, fitness is increased by having multiple responses (Fig. 2b). Different plastic traits will vary in how they interact with the environment because they also vary in characteristics such as their benefits, costs, and reversibility. If each response is more effective under different environmental conditions, plasticity in multiple traits will be favored. For example, different induced defensive responses often work better against different predators (Relyea 2004). Because it is ultimately the net benefit of a plastic response which matters, variation in the costs of the traits, including tradeoffs with other aspects of performance, can also favor plasticity in multiple traits if the importance of those costs varies among environments. Both synergy and complementarity can be tested by comparing the performance of individuals with different combinations of plastic traits in different environments (Box 3).

**Box 3: Testing the benefits of multivariate plasticity**

As with cue‐ and response‐mediated interactions, testing the fitness benefits of multivariate plasticity is best achieved through experimental manipulation of phenotypes. Specifically, testing the synergy and complementarity hypotheses relies on comparing the benefits and costs associated with different combinations of traits and environment, keeping in mind that the costs and benefits of the traits themselves, rather than plasticity per se, are typically most important for these hypotheses. To identify synergy, the performance of organisms with different combinations of plastic traits can be measured in the inducing environment, and then the combined performance of the responses compared to their independent performance (Fig. 2a). A positive statistical interaction indicates functional integration between the traits. For example, Van Buskirk and McCollum (2000) identified functional integration between tail morphology and activity level of *Hyla versicolor* tadpoles by using developmental conditions to manipulate morphology and then testing how the manipulated morphology and among‐individual variation in activity level jointly determined predation risk from predatory dragonfly larvae (Van Buskirk and McCollum 2000). Any case in which performance is higher for the combined response than either independent response indicates that having multiple plastic traits could overcome limits to changes in individual traits (a conclusion that can be further supported by identifying those limits).

Testing the complementarity hypothesis relies on identifying conditions under which each plastic response provides a greater net benefit, keeping in mind that this overall difference may depend on the benefits, costs, and reversibility of the plastic traits. Unlike synergy, complementarity is agnostic regarding the joint performance of the traits expressed together and can apply even if the responses interfere strongly with each other. Instead, these differences can be identified by exposing individuals to the different environmental conditions in which each response is hypothesized to perform best and measuring the consequences of variation in each trait. Although not essential for complementarity, distinct patterns of expression may also characterize complementary traits, where each plastic response is greatest under the conditions where it performs best (potentially indicated by a secondary cue). For example, an experiment in *Rana temporia* tadpoles that factorially manipulated predator cues and tadpole density supported complementarity: the behavioral response to predators was strong at low density and the morphological response was strong at high density (Teplitsky and Laurila 2007).

Experimental manipulation—although ideal for determining causation—will not always be tractable for all relevant factors. When a plastic trait cannot be independently manipulated, nonplastic trait variation (from genetics or other sources) can potentially substitute for plastic variation. This other variation should also cause many of the same effects as plastic changes in the trait, although the physiological changes underlying response‐mediated interactions may not be present. As another option, structural equation models (SEMs), which incorporate hypotheses about causation, can enable testing causal relationships when not all relevant factors can be experimentally manipulated. For example, Tonsor and Scheiner (2007) used SEMs to test plasticity in the effect of physiological and morphological traits on fitness via life history traits in *Arabidopsis thaliana*. SEMs may be particularly valuable for combining studies of development and selection in multivariate traits because the pathways through which traits effect both the development of other traits and fitness can be incorporated in the same model.

The plastic responses of tadpoles to predators provide evidence for both synergy and complementarity, albeit in studies of different species. Many tadpole species respond to predators in their ponds, typically dragonfly nymphs, with changes in morphology and behavior. When chemical cues from predators and injured tadpoles are present, tadpoles reduce their overall activity level to avoid detection, but they also develop broader, sometimes brightly colored tails which help to avoid predators by increasing escape speed and diverting predator attacks from the main body (McCollum and Van Buskirk 1996; Van Buskirk and McCollum 2000; Van Buskirk et al. 2003, Van Buskirk et al. 2004). Experiments with *Hyla chrysocelis* (gray treefrog) tadpoles indicate synergy between these responses. Tadpoles exposed to cues from tadpole‐fed predators developed the appropriate tail morphology and benefited more than tadpoles with noninduced tails when they reduced their activity in later predator encounters (Van Buskirk and McCollum 2000). Experiments with *Rana temporia* (common frog) tadpoles indicate complementarity between these responses, specifically across environments that differ in competition. In low‐competition environments, there is little cost to inactivity because of the greater availability of food, so tadpoles in these environments reduce activity more when exposed to predators. Alternatively, in high‐competition environments, the cost of inactivity is high, so tadpoles show a greater morphological response to predators instead (Teplitsky and Laurila 2007).

A special, but potentially common, source of complementarity occurs when plastic traits differ in their reversibility (also known as lability). Rates of environmental change vary extensively and are often multiperiodic. For example, weather conditions change in both daily and annual cycles. Similarly, plastic traits vary in how quickly reversible they are. In the previous example of foraging in *C. canutus*, a larger gizzard takes weeks to develop whereas behavioral changes in foraging‐site preference provide a rapid response which can be used as soon as the birds finish migration (van Gils et al. 2005). Slower, irreversible plasticity can be suitable for long‐term environmental change, especially when it occurs over multiple generations (Gabriel and Lynch 1992; Gabriel 1999). In contrast, frequent, within‐generation environmental change should favor fast, reversible plastic responses if all other benefits and costs are equivalent (Padilla and Adolph 1996; Gabriel 1999; Gabriel et al. 2005). If plastic traits vary in their reversibility, they can complement each other by being better suited for different rates of environmental change. In particular, slow but inexpensive plasticity in one trait can complement fast but expensive plasticity in a second trait when environmental change is multiperiodic by allowing the organism to achieve more accurate tracking of the environment with lower overall cost (Lande 2019).

Thermoregulation in the previously discussed *B. philenor* caterpillars provides an example of temporal complementarity in which two traits differ in reversibility. Color change and refuge‐seeking behavior occur on different timescales but also differ in cost. Although refuge‐seeking behavior provides a faster, stronger response to high temperatures, it can also be quite costly because caterpillars not on their host cannot eat, slowing their growth (Stockhoff 1998; Tammaru et al. 2004), and because caterpillars that leave their host risk not finding a host afterward (Rausher 1979). Color change allows *B. philenor* to stay on its hosts longer and avoid these costs of refuge seeking; however, caterpillars can only change color when they molt, which occurs at most once a day and often less frequently. Color change provides a more efficient response to long‐term (across‐day) temperature change while refuge seeking provides a more effective response to rapid (within‐day) temperature change, making this a case of temporal complementarity (Nielsen and Papaj 2017). Synergy, on the other hand, appears to be absent: the cooling effects of red coloration and a refuge position are largely redundant, with position having a much larger effect on operative temperature and refuge‐seeking alone being sufficient to ensure almost complete survival even at the hottest times of year (Nielsen and Papaj 2017).

## The Evolution of Plasticity in Multiple Traits: Implications and Future Directions

Understanding the above interactions in both the development of multivariate plasticity and selection on it can change how we think about classic topics in plasticity and evolution, including both how plasticity evolves and the evolutionary consequences of plasticity. An important first step will be understanding whether multivariate plasticity constrains or facilitates evolution. Overall, the evolution of multivariate plasticity will depend heavily on the relationship between plastic variation and genetic variation in those traits: recent meta‐analyses indicate that the direction of genetic variation and multivariate plastic change vary among studies but align more often than expected by chance, facilitating evolutionary change (Noble et al. 2019), but the direction of multivariate plasticity does not typically align with observed local adaptation (Radersma et al. 2020). The connection between multivariate plasticity and genetic variation should be stronger for traits with response‐mediated interactions—which should often rely on shared regulatory pathways that genes can also affect—than traits with cue‐mediated interactions—which are primarily a function of the environment instead of physiology. As with correlated traits more generally, the linked expression of plasticity in two traits can increase the rate at which the traits respond to selection, particularly when the traits show synergy (e.g., when they are functionally integrated; Ledón‐Rettig and Ragsdale 2021). On the other hand, when two plastic traits are complementary and function best under different conditions, selection may act to break any effect they have on each other's development. Responding to such selection, of course, requires genetic variation in the developmental interaction between traits (Ledón‐Rettig and Ragsdale 2021), which may not exist for some cue‐mediated interactions where the effect on the cue or environment is an inherent property of the phenotype itself (e.g., the effects of morphology on temperature).

Considering multivariate plasticity can also change how we think about the costs of plasticity. Most research based on plasticity in individual traits has argued that maintenance costs—also known as global costs and paid in all environments regardless of whether plasticity is used—are the most important costs of plasticity, while downplaying the importance of production costs—also known as local costs—which are only paid in environments where plasticity is used (Auld, Agrawal, and Relyea 2010). Because production costs are paid only when the benefit of plasticity is also gained, they are expected to have less effect on the evolution of plasticity than maintenance costs, which can select for reduction or even loss of plasticity (Sultan and Spencer 2002; Ernande and Dieckmann 2004; Auld, Agrawal, and Relyea 2010). Nevertheless, when multiple, independent plastic responses are available, production costs should also be important. If a plastic change has high production costs, but a less expensive alternative is available, selection should act to reduce its use, either by reducing overall plasticity in that trait or by increasing the threshold required to induce a change in the trait, a pattern that will not be the case for maintenance costs. Selection should particularly favor the reduced use of plastic responses with high production costs when other plastic responses have already occurred, potentially leading to the evolution of a response‐mediated interaction where a less costly plastic change alters the expression of more costly plasticity to reduce its use (i.e., serial developmental integration; Lande 2019). High production costs will also reduce the potential for synergy between traits by reducing the overall benefit of producing multiple responses, meaning that complementarity may be a more important factor in the evolution of plasticity when production costs are present.

In addition to changing how plasticity evolves, multivariate plasticity could also change the impact of plasticity on evolution. A key potential role of single‐trait plasticity in evolution is facilitating initial survival in a rapidly changing or novel environment; plasticity can reduce the risk of extinction and buy time for slower, evolutionary adaptation to the new environment (Ghalambor et al. 2007; Diamond and Martin 2021) as long as the initial plastic response is not perfect and without cost, which would negate the need for further evolution (Scheiner and Levis 2021). The ability of populations to persist and recover, however, depends on their overall mean fitness (Lande 2009), and thus depends not just on plasticity in a single trait but plasticity in all the traits that respond to the altered environment. It also then depends on how those traits interact to determine performance and fitness: the ability of plasticity to “buy time” should be greater for synergistic plastic traits because they will provide a greater overall fitness (i.e., demographic) benefit than when only the single trait changes. On the other hand, negative interactions between traits would lead to the overestimation of the ability to “buy time.” When plastic traits are complimentary, which trait is more important for facilitating evolution will depend on what aspect of the environment changes. Buying time via phenotypic plasticity is particularly important when considering the impacts of contemporary anthropogenic changes such as climate change or urbanization (Diamond and Martin 2021). To fully understand whether existing plasticity will be sufficient to prevent extinction and enable adaptation, we need to consider the full range of plastic responses available and how they interact with each other.

## Conclusions

Here, we have highlighted examples of different ways that adaptive plasticity in multiple traits can interact both during the development of those traits and later selection on the traits. Few studies have directly investigated the hypotheses outlined here regarding how plastic traits can change each other's expression or the fitness benefits of having multiple plastic responses; even fewer have tested their evolutionary implications. Our examples demonstrate how careful, manipulative experiments can be used to test the causation of interactions and address these hypotheses. Incorporating multiple plastic traits and their interactions into existing models of plasticity and its evolution (e.g., Scheiner 2018, Lande 2019) has also proven a productive starting point for generating new hypotheses regarding these interactions and their evolutionary consequences. Directly testing these hypotheses in additional taxa and environmental contexts should be a priority for future research. Fully understanding interactions among plastic traits, either during development or selection, will often require combining the results of multiple studies. We did that here for multiple examples focused on morphology and behavior, but more may exist elsewhere in the literature, and even more are yet untested. In particular, we have not yet identified clear examples of response‐mediated interactions, but these may be more common among other types of traits. Regardless, we have shown here that plastic responses in different traits can interact in ways that would go undetected if we only studied the plasticity of single traits in isolation. To understand phenotypic plasticity fully, we must consider it in the context of the entire organism, which can often respond to its environment in many ways. So far, we have only scratched the surface of how plastic traits can interact, and we will surely gain many new insights as we further study the causes and consequences of these interactions.

## AUTHOR CONTRIBUTIONS

MEN drafted the manuscript with assistance from DRP. Both authors approve of the final submission.

## CONFLICT OF INTEREST

The authors declare that there is no conflict of interest.

Associate Editor: Dr. Matthew R Walsh

Handling Editor: Prof. Tracey Chapman
